# Construct, Face, and Content Validation on Voxel-Man® Simulator for Otologic Surgical Training

**DOI:** 10.1155/2017/2707690

**Published:** 2017-05-03

**Authors:** M. Varoquier, C. P. Hoffmann, C. Perrenot, N. Tran, C. Parietti-Winkler

**Affiliations:** ^1^Department of Oto-Rhino-Laryngology, Head and Neck Surgery, University Hospital of Nancy, 29 Av. de Lattre de Tassigny, 54000 Nancy, France; ^2^Développement, Adaptation et Handicap Laboratory (DevAH-EA 3450), Faculty of Medicine and Faculty of Sports Science, University of Lorraine, 54500 Vandœuvre-lès-Nancy, France; ^3^Department of General and Emergency Surgery, University Hospital of Nancy, Av. de Lattre de Tassigny, 54000 Nancy, France; ^4^The Nancy School of Surgery, Faculty of Medicine, University of Lorraine, 54500 Vandœuvre-lès-Nancy, France

## Abstract

*Objective*. To assess the face, content, and construct validity of the Voxel-Man TempoSurg Virtual Reality simulator.* Participants and Methods*. 74 ear, nose, and throat (ENT) surgeons participated. They were assigned to one of two groups according to their level of expertise: the expert group (*n* = 16) and the novice group (*n* = 58). The participants performed four temporal bone dissection tasks on the simulator. Performances were assessed by a global score and then compared to assess the construct validity of the simulator. Finally, the expert group assessed the face and content validity by means of a five-point Likert-type scale.* Results*. experienced surgeons performed better (*p* < .01) and faster (*p* < .001) than the novices. However, the groups did not differ in terms of bone volume removed (*p* = .11) or number of injuries (*p* = .37). 93.7% of experienced surgeons stated they would recommend this simulator for anatomical learning. Most (87.5%) also thought that it could be integrated into surgical training.* Conclusion*. The Voxel-Man TempoSurg Virtual Reality simulator constitutes an interesting complementary tool to traditional teaching methods for training in otologic surgery.

## 1. Introduction

Temporal bone surgery requires thorough knowledge of middle-ear anatomy and great surgical precision. Novice surgeons typically obtain anatomical knowledge by means of anatomical boards, yet these do not offer them a good three-dimensional (3D) visual representation of the middle-ear's structures. In addition, until recently, the only way for beginners to acquire otologic skills was to train on cadaveric models due to the absence of any adequate animal model. However, obstacles imposed by legislative and ethical issues, as well as costs, seriously limited access to human temporal bones [1], thus reducing the total training time [2]. New technologies could help overcome these limitations by developing innovative methods for teaching complex otologic skills.

Virtual reality (VR) is of growing interest in medical education, especially in surgery [3–5]. This new tool offers many advantages over traditional forms of learning by teaching technical skills in a controlled and safe environment, ensuring reproducible conditions. In addition, novice surgeons can practice independently, unhindered by constraints: material support, availability of expert tutors, and operating time. More importantly, VR simulators allow medical students to repeatedly practice a series of standardized surgical tasks until the gesture is perfectly executed. This process reduces the risks for both patients and students. This is crucial in surgery, where even a minor error can lead to dramatic consequences for the patient.

Before it can be used in a training program, a simulator must fulfill several criteria. The principles of validating surgical simulators are based on two aspects [6]: subjective and objective validity. Subjective validity is assessed by the face and content validity, which are usually evaluated by satisfaction surveys completed by experienced surgeons. Face validity measures the realism of various components and aspects of the simulator, whereas the content validity assesses the pedagogical value and training effectiveness of the simulation platform [6]. Objective validity is largely concerned with the construct validity, which refers to the simulator's ability to distinguish between different levels of expertise [7].

The Voxel-Man TempoSurg simulator was the first commercially available temporal bone simulator and has already been evaluated a number of times in the literature [8–14]. In 2012, Arora et al. [10] assessed both its face and content validity and suggested that the simulator's realism could be improved. However, they considered the opinions of both expert and novice surgeons, whereas it is recommended to evaluate the subjective validity of a simulator using only experts' assessments [6]. With regard to the construct validity, Khemani et al. [12] reported that the objective metrics produced by the simulator can be used to differentiate between surgeons of differing levels of expertise. This result is in line with that reported by Linke et al. [8], where experienced ear surgeons performed better and faster than residents and students. However, no differences were found between the groups about bone volume removed. In both studies, they evaluated surgeons on one particular simulated surgical task whereas the simulator could offer a substantial list of different exercises. We therefore focused our attention in this study on four temporal dissection tasks of fundamental importance in ear surgery, as they correspond to the first steps of ear surgery learning. Through testing the latest version of the simulator, we aimed to assess the face, content, and construct validity of the Voxel-Man TempoSurg VR simulator for temporal bone dissection in an otologic training program.

## 2. Material and Methods

### 2.1. Participants

74 ear, nose, and throat (ENT) surgeons took part in this experiment (Table 1) coming in from all over France. They were divided into two groups according to their level of expertise: the expert group (≥50 mastoidectomies performed, *n* = 16) and the novice group (<50 mastoidectomies, *n* = 58). Participants of the novice group were recruited during training sessions held at the Nancy School of Surgery, France. Experts were asked directly (i.e., telephone, mail) whether they would accept to take part in the study. All participants were informed about the aims of the study and each provided informed consent prior to participation in the study.

### 2.2. Simulator

**Figure 1:**
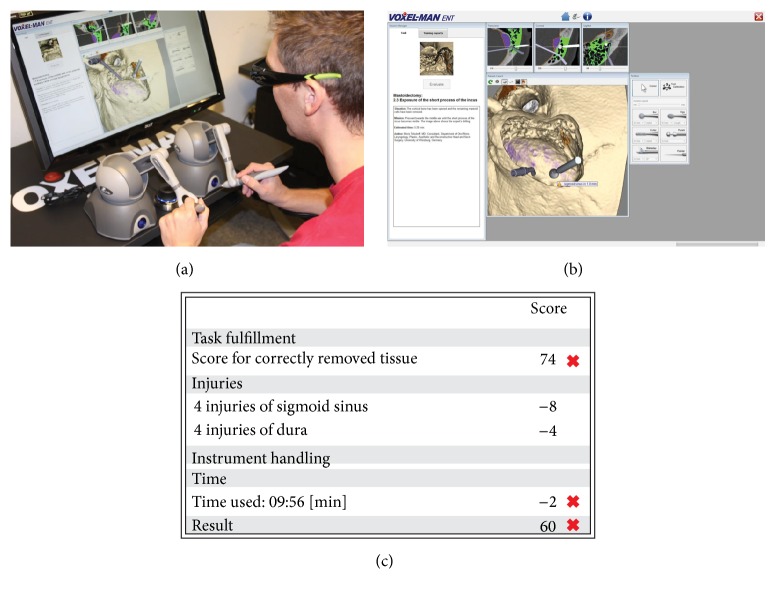
(a) Experimental design, (b) first-person view, and (c) automatic performance metrics generated by the Voxel-Man TempoSurg simulator.

All testing was performed on a Voxel-Man TempoSurg simulator (Voxel-Man, Hamburg, Germany) at the Nancy School of Surgery, France. The participants were seated at a table in front of a 3D monitor, which was used to display a virtual environment that simulated a right-ear surgery (Figures 1(a) and 1(b)). The image on the screen was similar to what participants would experience during surgery. The skull, derived from high-resolution computed tomography (CT) data, and instruments were modeled in high-definition by a computer. The instruments were controlled using force-feedback hand styluses, one for each hand, which could be moved in all three dimensions. The drilling function was activated by a foot pedal and the simulator provided a large selection of metal and diamond burs of different shapes. The surgical site could be inspected from all possible directions at any point. The simulation was continuously updated by the participant's interaction with the two force-feedback devices. At the end of each task, the simulator provided a global score based on the bone volume removed and penalties for injuries of structures at risk and exceeding time limits. An optimal volume of 100% was established by the simulator, which was compared to the participant's bone volume removed (Figure 1(c)). 100% of bone removed gives 100 points to the participant. This is the highest score. Then, the simulator gave penalties if participants injured any anatomical structures. Simulator counts an injury when the drill touched a structure (e.g., dura, sigmoid sinus, ossicular chain, and facial nerve). Each penalty was weighted according to the severity of injury (e.g., 1 point for dura injury, 20 points for facial nerve injury, and 50 points for brain injury). Finally, a 1-point penalty was applied to the global score for each minute exceeding the time limit.

### 2.3. Experimental Design (Figure 2)

The novice surgeons were provided with a standardized anatomic description and basic surgical skills by a confirmed surgeon. Both groups were then presented with a standardized description of the simulator, including its composition and working mode. Before starting the evaluation, each participant performed a simple 5-minute drilling task, writing their names, in order to familiarize themselves with the simulator. Once the familiarization phase was completed, each participant performed only once the following four temporal bone dissection tasks in standardized conditions (i.e., normal right ear): opening the cortical bone, exposure of the sigmoid sinus, exposure of the short process of the incus, and exposure of the horizontal semicircular canal. These four tasks were selected from the list offered by the simulator for their educational value. The participants were instructed to perform each task as fast as possible without damaging the neighboring anatomic structures. Once all the dissection tasks were completed, the realism and training effectiveness of the simulator were assessed by the expert group using qualitative surveys rated on a five-point Likert-type scale, in which 1 represented not true/realistic/useful and 5 represented very true/realistic/useful. A score of 3 was considered neutral.

### 2.4. Data Acquisition

The outcome measures for each participant in each dissection task were global score, bone volume removed, time taken, efficiency (i.e., bone volume removed per second), and number of structure injuries obtained from the Voxel-Man simulator. The groups' performances were compared to assess the construct validity of the simulator. Data from the survey was collected to assess face and content validity.

### 2.5. Statistical Analysis

The Statistical Package for the Social Sciences (SPSS) Version 20.0 (SPSS, Inc., an IBM Company, Chicago, Illinois) was used for analyses. All data was examined for normality using the Shapiro-Wilk test. Due to the nonnormal distribution of data, a nonparametric Mann-Whitney* U* test was used to compare the overall performances of the expert and novice groups, consisting of global score, bone volume removed, time taken, efficiency, and number of structure injuries. Another nonparametric Mann-Whitney* U* test was conducted to compare between performances of the two groups in each temporal bone dissection task. All data were reported as median (interquartile range (IQR)) within the text. The level of significance was set to *p* < .05.

## 3. Results

### 3.1. Construct Validity (Figure 3)

When we consider all of the four tasks, our results demonstrated that the experts globally scored better than the novices (*z* = 3.03, *p* < .01). Univariate analysis showed that the experts globally performed faster than the novices (*z* = 4.84, *p* < .001). Both groups globally removed the same volume of bone (*z* = 1.60,   *p* = .11). In addition, the experienced surgeons were more efficient than the students (*z* = 5.64, *p* < .001). The novices injured the same number of structures as the experts when considering all the tasks (*z* = 0.90,   *p* = .37), with a more detailed analysis revealing that they only inflicted more injuries on the posterior canal wall, in comparison with the experts (*z* = 3.29,   *p* = .001) (Table 2).

### 3.2. Subjective Validity

#### 3.2.1. Face Validity

The realism of the simulator was assessed across several items: the appearance of the drill and anatomical structures, evaluation of the drill-handling characteristics, and quality of the graphics (Table 3). The global realism assessment score was satisfactory (mean score: 3.4). All except one experienced ear surgeon rated the simulator as realistic (mean score: 3.4). Only the ergonomics of the simulator were rated below average (mean score: 2.5). All the other items were judged as acceptable and the most satisfying were the realism of the anatomic structures and drill (4.3 and 3.9, resp.).

#### 3.2.2. Content Validity

The degree to which the simulator represented the fundamental problem of a temporal bone dissection was assessed across the following items: surgical anatomy and planning, drill navigation and technique, hand-eye coordination, and overall usefulness as a training tool (Table 4). All the experts, except one, positively rated the simulator (mean score: 4.1), in particular noting the pedagogical value of the simulator (mean score: 4.1). In addition, 93.7% of the experienced surgeons said they would recommend the Voxel-Man simulator for anatomy learning (score of at least 4 out of 5). Most of them (87.5%) also thought that this simulator could be integrated into surgical training courses (score of at least 4 out of 5).

## 4. Discussion

In this study, we sought to assess whether the Voxel-Man simulator can distinguish between experienced and novice surgeons. To meet this objective, we selected four temporal bone dissection tasks considered as the first fundamental steps in most middle- and inner-ear surgeries. Our results demonstrate that experienced surgeons obtained better overall scores than novices. In addition, the experts completed the tasks faster while removing the same volume of bone. These results support the construct validity of the Voxel-Man TempoSurg VR simulator. Previous studies have supported the construct validity of the Voxel-Man TempoSurg simulator for a specific task [8, 12]. We here divided the mastoidectomy into four stepwise tasks, as it remained unclear whether the validity of the simulator was transferable to an entire procedure like inner- and middle-ear surgery. It appears interesting to test novice surgeon through a whole logical surgical procedure, more than to individual not-related tasks, to identify his/her possible weaknesses that could be masked by a global score comparable to that of experts.

Previous studies have identified the time taken and number of injuries as key factors in differentiating surgeons of differing levels of expertise [15–17]. Although our data revealed that the experienced surgeons took less time to complete the tasks, they did demonstrate that the experts caused injuries to structures at risk as frequently as novices, except for the posterior wall of the outer ear canal, which was less often injured by the experts. This could be on account of some structures (e.g., the dura or sigmoid sinus) being used by surgeons as surgical landmarks. Furthermore, they did not hesitate to come in contact with them with the drill to distinctly identify areas at risks. The exposure of critical structures calls for extreme precision and extensive knowledge of ear anatomy. Despite our analyses revealing no significant differences in the number of injuries caused between the groups, we can see that the novices more often tended to injure all the structures. This may reflect a lack of anatomical knowledge or be related to excessive force exerted on the drill. In this regard, Zhao et al. [18] reported that expert surgeons tend to start virtual temporal bone surgery by exerting high force and then reducing this force when approaching critical structures, contrary to novices who used the same force throughout the task. This result points out the importance of having a thorough knowledge of the location of each structure. For instance, the external auditory canal wall has to be cleared, without harming it, in order to avoid injuries to the facial nerve, which is very close. The lack of knowledge and experience among novices could explain why they touched this structure more often than the experts. Interestingly, in our study, the experts never injured fundamental structures like the facial nerve or chorda tympani. This supports the observation that experts are less hesitant in approaching very close to structures at risk or even in touching them, according to their importance.

Other variables could also be added for greater assessment of expertise. For instance, Khemani et al. [12] found that experienced surgeons removed larger bone volumes compared to novices. However, Linke et al. [8] reported no differences between their participants regarding this variable. In line with the latter, we here found no differences, globally, in the bone volume removed by experts and that removed by novice ear surgeons. These results suggest that experienced surgeons are able to perform efficient middle-ear surgery without removing large volumes of bone. The ratio of bone volume removed by the participant to reference volume therefore appears to represent an inappropriate variable as the most important criterion for assessing task achievement, which is exactly what the software included with the Voxel-Man TempoSurg simulator does. As previously mentioned, the time taken by participants to complete the different tasks appeared to be a more discriminative criterion than this ratio, as did the drilling efficiency of surgeons. Still, the objective metrics generated by the simulator seem to be suboptimal and could be improved by according more weight to other key factors of performance, such as the time taken or drilling efficiency, while taking into account injuries to critical structures.

The simulator obtained an overall good appreciation for face and content validity. All the experts particularly appreciated the anatomical disposition of the structures and drill running. Most of the experienced surgeons (93.7%) said they would recommend the Voxel-Man simulator for teaching ear anatomy and thought that this simulator could be integrated into surgical training courses. These results are in line with previous studies assessing the face and content validity of the Voxel-Man [9, 10]. In a recent study, Arora et al. [19] suggested that the Voxel-Man could be included in a proficiency-based curriculum. They evaluated the realism of the simulator as suboptimal and encouraged the manufacturer to improve the drill ergonomics and deepen the visual-spatial perception. For a current simulator, we attest that the Voxel-Man TempoSurg provides a satisfactory resemblance to the anatomic details of the human temporal bone. It allows students to expand their knowledge of temporal bone anatomy and to perform surgical tasks with relatively realistic haptic feedback. Nevertheless, some features do require improvement, such as the ergonomics. However, the issue then arises of whether an absolutely perfect reproduction of human temporal bone surgery is truly necessary to acquire strong surgical otologic skills. Further work should be conducted in consideration of this issue by investigating the effectiveness of the simulator as a learning tool, in which an assessment of learning curves could be interesting. In addition, the performance of inexperienced ear surgeons over time could be compared to those of experts to determine at which time point there is no longer any difference between the two groups. One other interesting perspective could be to consider the transferability of surgical otologic skills learned with simulator to real-life surgery. Lastly, one could also query whether the VR simulator could be used for the certification of medical students, who would not be authorized, for example, to operate on real patients before having reached a certain number of points on the simulation platform.

Real-life surgery training consists of the teaching and supervision of a more or less inexperienced surgeon by an experienced ear surgeon. However, the presence of an expert does not reduce the risk of medical error to zero. Moreover, the operational time tends to be increased by the instructions and advice given by the mentor to his student. Longer operation times can induce more postoperative complications for patients and increase costs for hospitals. These issues underline the need to develop alternative methods of learning outside the operating room. Training using a virtual temporal bone offers many significant advantages, including the possibility of training on normal or pathological ears and of repeating the procedure an infinite number of times in reproducible conditions. Simulators are accessible, allowing students to self-correct their errors by starting over, providing the possibility of self-assessment, and reducing the consumption of cadaveric material. Temporal bone surgery is complex, with small errors in judgment resulting in potentially dramatic consequences [20]. It requires extensive surgical skills that training on a simulator can provide. Experienced surgeons could also use VR to prepare for specific surgeries with special surgical conditions in order to minimize patient risk. Nevertheless, despite technological advances, current virtual surgical simulators cannot replace traditional human temporal bone surgery, yet they can supplement traditional training methods for ear surgeons.

## 5. Conclusion

The Voxel-Man TempoSurg Virtual Reality simulator constitutes an interesting complementary tool to traditional teaching methods for training in otologic surgery. Although some features require improvements, this simulator allows trainees to acquire a good 3D visualization of ear structures and to learn complex surgical skills. The simulator's ability to distinguish between different levels of expertise largely depends on the tasks submitted to participants. Thus, by selecting appropriate exercise, this simulator could also be used as a certification tool, constituting a prior condition for performing real-life surgery.

## Figures and Tables

**Figure 2:**
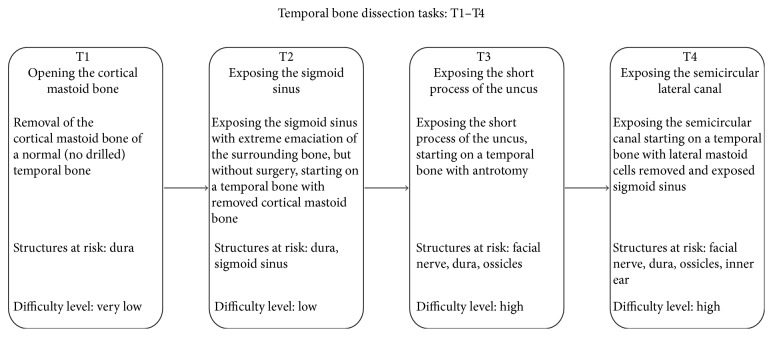
Illustration of the experimental protocol.

**Figure 3:**
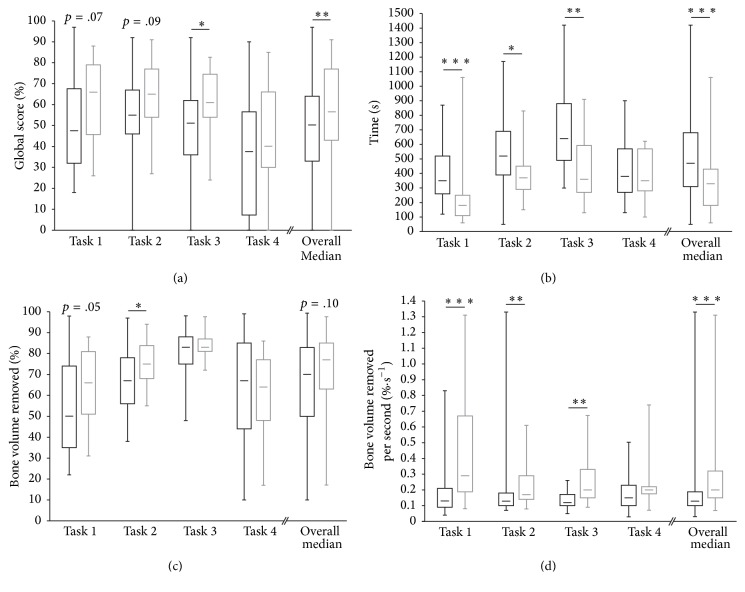
(a) Median global score, (b) time, (c) bone volume removed, and (d) the ratio of bone volume removed per second for the novice group (white bar) and expert group (grey bar). ^*∗*^Significant difference between the two groups (^*∗*^*p* < .05, ^*∗∗*^*p* < .01, and ^*∗∗∗*^*p* < .001).

**Table 1:** Demographic characteristics of participants.

	All participants	Novice group	Expert group
	*n* = 73	*n* = 58	*n* = 15
Mean age (years)	30.3 ± 9.1	26.8 ± 2.0	45.8 ± 12.0
Gender, *n* (%)			
Male	38 (52%)	27 (47%)	11 (73%)
Female	35 (48%)	31 (53%)	4 (27%)
Dominant hand, *n* (%)			
Right-handed	63 (86%)	48 (83%)	15 (100%)
Left-handed	10 (14%)	10 (17%)	0 (0%)

**Table 2:** Injuries of structures at risk.

	Novice group	Expert group
	*n* = 58	*n* = 15
Total injuries	894	125
	9 (5.25–15.75)	6 (4.5–11)
Injury of the dura	175	45
	1.5 (1–3.75)	3 (1.5–4)
Injury of the sigmoid sinus	81	30
	0.5 (0–2)	1 (0–2.5)
Injury to the brain	20	9
	0 (0-0)	0 (0-1)
Injury of the auditory ossicles	218	37
	2.5 (1–5.75)	2 (1–3.5)
Injury of the vestibular labyrinth	65	2
	0 (0-1)	0 (0-0)
Injury of the chorda tympani	2	0
	0 (0-0)	0 (0-0)
Injury of the facial nerve	8	0
	0 (0-0)	0 (0-0)
Injury of the posterior wall of the outer ear canal^*∗*^	325	2
	1 (0–3)	0 (0-0)

Results represent occurrence and median (interquartile range).

*∗* indicates significant differences (*p* < .01) between the expert and novice groups.

**Table 3:** Face validity.

	Expert group
	*n* = 16
Global assessment	3.4 ± 1.1
Appearance of anatomical structures	3.5 ± 1.0
Appearance of anatomical rapports	4.3 ± 0.6
Appearance of drill	3.6 ± 0.7
Controlling of drill	3.1 ± 1.1
Haptic feedback	3.1 ± 1.0
Performance of drill	3.9 ± 0.7
Ergonomics	2.5 ± 1.2

Data is presented mean ± standard deviation.

**Table 4:** Content validity.

	Expert group
	*n* = 16
Global assessment	4.1 ± 1.1
Teaching anatomy	4.7 ± 0.5
Teaching surgical planning	4.1 ± 1.3
Training hand-eye coordination	3.9 ± 1.1
Curriculum	4.5 ± 0.8
Transfer to operating room	3.1 ± 1.5

Data is presented as mean ± standard deviation.
